# Editorial: Juvenile Spondyloarthritis: From Basic Science to Clinical Translation

**DOI:** 10.3389/fmed.2022.861512

**Published:** 2022-03-08

**Authors:** Miroslav Harjacek, Rik Joos, Ruben Burgos Vargas

**Affiliations:** ^1^Department of Pediatrics, College of Medicine and Health Sciences, United Arab Emirates University, Al Ain, United Arab Emirates; ^2^Department of Rheumatology, Ziekenhuisnetwerk Antwerpen, Antwerp, Belgium; ^3^Pediatric Rheumatology, University Hospital, Gent, Belgium; ^4^General Hospital of Mexico, Mexico, Mexico

**Keywords:** juvenile spondyloarthritis (JSpA), classification, translational science, pathophysiology, therapy, biomarkers, outcome

## Introduction

Juvenile spondyloarthritis (JSpA) concept emerged relatively recently. Although through many years, attempts have been made to define this group of diseases, no uniform consensus could be reached. In a recent advanced course endorsed by Pediatric Rheumatology European Association PReS, in 2019. Authorities from all over the World joined in Zagreb, Croatia to exchange points of view and most recent knowledge. This Special Issue is the final result of this continuous effort of both rheumatologists and pediatric rheumatologists to grasp on JSpA. Without the close collaboration between these two specialties the long ongoing discussions and even misunderstandings would remain. Through a series of articles authors underscore the recent progress in our knowledge of JSpA classification, pathophysiology, diagnosis, similarities, and differences between adult and juvenile forms and treatment. It also pinpoints to unmet needs in translational research (e.g., biomarkers) to improve suboptimal outcomes in JSpA.

Juvenile spondyloarthritis is an umbrella term for a group of inflammatory diseases that involve the spine (sacroiliitis and spondylitis), joints (asymmetric peripheral arthritis), and tendons (enthesitis). Juvenile spondyloarthritis is now considered as a distinct subtype of juvenile arthritis (JIA). It is characterized by male predominance and later onset in childhood. Juvenile ankylosing spondylitis (jAS), reactive arthritis, juvenile psoriatic arthritis (JPsA), arthritis associated with inflammatory bowel disease (IBD), and undifferentiated JSpA (u-JSpA) or enthesitis related arthritis (ErA) are all fitting into the JSpA concept. While spondyloarthritis (SpA) in general, is one of the most widespread chronic rheumatic diseases, JSpA is also seen globally. Enthesitis related arthritis is the most common at about 10–40%, while JPsA represents ~2 to 10% of all JIA patients and jAS, as the most severe form, is seen in about 1–7% of JIA children worldwide (1). The diagnosis of JSpA can often be difficult because the symptoms are sometimes episodic and unpredictable and the initial presentation can be subtle. Main symptoms include a peripheral asymmetrical arthritis, frequently hip involvement, and enthesitis as a hallmark of the disease. Spine disease is rare, and it may take several years for axial disease to develop in children. The HLA-B27 positivity is well-known as a risk factor for axial disease, but is not diagnostic for JSpA. Many children diagnosed with JSpA will eventually fulfill criteria for adult SpA suggesting common clinical patterns and pathophysiology. Similarly, to adults, when compared to healthy controls, recent studies have shown clear alterations in gut microbiome in patients with JSpA. It appears convincingly that innate immune system, and in particular, interleukin 17A (IL-17/23 axis) are playing a pivotal role in JSpA pathophysiology. No pathognomonic laboratory biomarker(s) for the disease(s) diagnosis exists, and the radiographic findings are often negative or illusive in the children with growing skeletons. Treatment of peripheral arthritis usually includes non-steroidal anti-inflammatory drugs, and joint steroid injections. Juvenile patients are virtually unresponsive to MTX treatment, whereas TNF inhibitors are the main biologic therapy for treatment of axial disease and conventional therapy resistant peripheral disease. Although other biologics such as IL-17 blockers are beneficial in adult SpA, none are currently approved for the use in children. Yet, studies in adults, and recent JUNIPERA study in children with ErA and JPsA are promising (2).

With fewer than half of children achieving remission off medication 5 years after diagnosis and hence, rapid identification and early treatment is paramount (3, 4). Following revised (Edmonton 2011) ILAR criteria patients are most often classified as ErA, JPsA, or unclassified JIA (5). Presently pediatric rheumatologists do not diagnose JPsA in all children whose disease signs and symptoms fulfill adult classification criteria for Psoriatic Arthritis (CASPAR), the unification of CASPAR and pediatric PsA classification criteria is needed (6).

## Overview of the Issue

In the article by Joos author tries to illustrate the way of thinking about JSpA in recent decades. Starting from the criteria formerly used in adult rheumatology, gradually the concept of JSpA is becoming clearer. A clear description of the characteristics by which a patient can be considered as suffering from a JSpA is essential to achieve reproducible research.

The similarities and differences between adult and juvenile form of SpA are outlined in the article by Fisher et al. As they emphasize, adult SpA and JSpA share many common signs and symptoms, implying that they are a spectrum of the same disease. Yet, the authors noticed main differences in classification criteria, clinical picture, disease activity, and pathophysiology. In particular, section on the effects of adolescence, sheds additional light on the role of sex hormones and mechanical stress on development of JSpA.

In his “Out of box view” author (Harjacek) offers his understanding of epigenetics, various stressors exposure, neuroendocrine pathways, and macrophage migration inhibitory factor (MIF) in the immunopathophysiology of jSpA. In affected patients subclinical gut inflammation, initiated by intestinal dysbiosis, is essential to the prospective development of inflammation in the synovial–entheseal complex. Although the crucial role of IL17/23 axis, TNF-α, and IL-7 in the pathophysiology of SpA, including JSpA, is well-known, the role of MIF is generally ignored, but recently validated (7, 8). The strong correlation of osteitis with low-grade IBD, established in children with ErA by elevated concentration of fecal calprotectin (fCAL) (Lamot et al.), emerges as the central event in the early stages of SpA.

Furthermore, in the review by Huan Tay et al. progresses in understanding the role of HLA-B27, development of enthesitis and new bone formation in JSpA pathophysiology and therapy, were critically discussed. As authors emphasize, enthesitis as the pathognomonic sign of SpA, caused mainly by exaggerated response to biomechanical stress, emerges paradoxically as osteoproliferation at inflamed periarticular sites in a setting of increased systemic bone resorption. It is still unclear if inflammation and osteoproliferation are directly linked or separated events.

Children with JSpA often present with extra-articular manifestations as is shown in the retrospective, observational, monocentric study of 53 ErA patients followed at the Mayer Childrens Hospital of Florence, Italy (Pagnini et al.). The authors hypothesized the existence of two distinctive disease phenotypes in children with ERA, whether extra-articular manifestations are present or absent. In particular, tarsitis was associated with ErA diagnosis and the absence of extra-articular manifestations. The development of extra-articular manifestations over period of time, might evolve to different disease, such as Crohn disease or SAPHO syndrome. Authors concluded that older age of disease onset, HLA-B27 positivity, development of the hip arthritis within the first 6 months and tarsitis, are all risk factors associated with worse prognosis of ErA patients.

Along the same lines, Romero-López et al. discuss the clinical and translational features of inflammatory foot involvement in SpA. The authors highlight the predominance of tarsal affection in JSpA patients among several cohorts. The most severe form of this disease has been named Ankylosing Tarsitis (AT). This review provides a proposal for classifying the clinical features in three stages (prodromal phase, disease continuum, and bone ankylosis) that differ in clinical and radiographic findings. Few reports are approaching the specific pathogenesis of tarsal inflammation and ankylosis. Many experimental models of SpA can start with midfoot inflammation and progress to axial disease, somehow resembling the clinics of young patients with SpA. Foot enthesitis and ossification are usually neglected, although there is an increasing evidence of its relevance as an initial clinical manifestation that can either alert about future progression, or act as a more accessible source of tissues or cells for translational studies.

The search for reliable and prognostic biomarkers in JSpA has been extensive but results so far have been disappointing. In the single center study by Lamot et al., the role of fCAL, a proposed biomarker of gut inflammation, was evaluated in 71 JIA patients. In patients with ERA, moderate correlation was detected between fCAL concentration and the disease activity measured by JSpADA. Moreover, fCAL concentration was able to discriminate between ErA patients with inactive or active disease. Contrary to some previous studies, the fCAL levels were not linked to the use of NSAID, nor with the use of other therapy for JIA. Intriguingly, ErA patients with signs of SIJ inflammation detected by contrast MRI, had a significantly higher fCAL concentration than those without the signs of SIJ involvement, suggesting that gut inflammation might be a integral part of a systemic inflammation existing in patients with ErA. Authors concluded that even in asymptomatic ErA patients with increased levels of fCAL, performing a contrast MRI of SIJ, might be helpful.

The real-life data, opposed to the data collected from the clinical trials are generally more useful for the daily clinical practice. Important questions about tapering/withdrawing classical DMARDs (cDMARDs) and biological therapy, in 75 ErA patients, were addressed in the retrospective study by Liao et al. Authors reached several important conclusions. Firstly, patients with a longer time interval between disease onset and initiation of cDMARDs had a higher risk of flare-ups during tapering of biologics. Secondly, they also disclosed that patients who had a shorter time to achieve clinical inactive disease once biological agents were started, seemed to have a lower chance of experiencing flare-up during biologics tapering. Thirdly, they confirmed that ANA positivity was present more frequently in patients under biologics, as previously reported. Due to insurance requirements Etanercept was the most commonly used first-line biologics in their cohort, yet showing less efficacy in those patients with higher risk of IBD development (9).

The focus of the final review in the series was the clinical burden of disease, prognostic indicators and outcomes in JSpA (Smith and Burgos-Vargas). Reviewing a number of articles regarding the clinical picture, and the data that allows the differentiation from other forms in the classification of JIA. The authors retake issues regarding the concept and nomenclature of SpA. The way this condition has been called throughout the years depends on who was the physician seeing the case. The indicial presentation to the pediatrician is surely peripheral arthritis and enthesitis and some years later, axial symptoms. Specialists will search for sacroiliitis and spondylitis and their differentiation from other forms of JIA. In reviewing the series of cases or the few cohort studies, investigators dedicated to adult onset Spa neglect the juvenile onset categories. In contrast, the pediatric rheumatologist tends to concentrate on the search of axial arthritis even in children and adolescents who have no evidence of sacroiliitis. Studies showing a transition in patients with JSpA toward adult forms confirm common clinical and genetic characteristics. The chapter approaches the relatively scarce information on the consequence of a disease, relatively rare, yet devastating. Therefor early recognition either by the presence of characteristic peripheral involvement or inflammatory back pain and sacroiliitis is needed.

## Summary

Through this Research Topic efforts have been made to precisely classify and describe the pathophysiology and clinical features of JSpA. In order to accomplish unmet clinical needs, early diagnosis and treatment of children with JSpA is essential. The search for more sensitive and specific biomarkers is ongoing. Customized therapy for each and every patient should improve outcome as an ultimate goal, and diminish unfavorable consequences of the disease burden for those children.

## Author Contributions

MH wrote initial version. RJ and RV wrote revisions and then MH submitted the final version. All authors contributed to the article and approved the submitted version.

## Conflict of Interest

The authors declare that the research was conducted in the absence of any commercial or financial relationships that could be construed as a potential conflict of interest.

## Publisher's Note

All claims expressed in this article are solely those of the authors and do not necessarily represent those of their affiliated organizations, or those of the publisher, the editors and the reviewers. Any product that may be evaluated in this article, or claim that may be made by its manufacturer, is not guaranteed or endorsed by the publisher.
